# The prevention of endothelial dysfunction through endothelial cell apoptosis inhibition in a hypercholesterolemic rabbit model: the effect of L-arginine supplementation

**DOI:** 10.1186/1476-511X-7-27

**Published:** 2008-08-02

**Authors:** Mehdi Nematbakhsh, Shaghayegh Haghjooyjavanmard, Farzaneh Mahmoodi, Ali Reza Monajemi

**Affiliations:** 1 Deparment of Physiology, Isfahan University of Medical Sciences, Isfahan, Iran; 2 Applied Physiology Research Center, Isfahan University of Medical Sciences, Isfahan, Iran

## Abstract

**Background:**

The impact of L-arginine on atherogenesis and its ability to prevent endothelial dysfunction have been studied extensively during the past years. L-arginine is a substance for nitric oxide synthesis which involves in apoptosis. Hypercholesterolemia promotes endothelial dysfunction, and it is hypothesized that L-arginine prevents endothelial dysfunction through endothelial cells apoptosis inhibition. To test this hypothesis, thirty rabbits were assigned into two groups. The control group received 1% cholesterol diet for 4 weeks, and the L-arginine group received same diets plus 3% L-arginine in drinking water.

**Results:**

No significant differences were observed in cholesterol level between two groups, but the nitrite concentration in L-arginine group was significantly higher than other group (control group: 11.8 ± 1; L-arginine group: 14.7 ± 0.5 μmol/l); (*p *< 0.05). The aorta score of fatty streak in control group was 0.875 ± 0.35, but no fatty streak lesion was detected in L-arginine group (*p *< 0.05). The number of intimal apoptotic cells/500 cells of aorta in two groups of experiment were statistically different (control group: 39.3 ± 7.6; L-arginine group: 21.5 ± 5.3) (*p *< 0.05).

**Conclusion:**

The inhibition of endothelial cells apoptosis by L-arginine restores endothelial function in a model of hypercholesterolemia.

## Background

The concept of programmed cell death was introduced to describe cell death during normal development [1], and apoptosis is the most common form of cell death. Apoptosis is characterized by cell shrinkage, nuclear fragmentation and membrane blabbing [2,3].

Atherosclerotic lesions develop in the tunica intima of the arteries, in which accumulation of cellular components, lipids, and extracellular matrix yields a fibro fatty plaque that focally thickens the artery wall [4]. Apoptosis is a feature of human atherosclerosis which is associated with development of the lesion necrotic core as well as instability of complex plaques [4-9].

The first evidence that endothelial cell (EC) apoptosis might contribute to the initiation of atherogenesis came from the observation that all classical risk factors known to promote endothelial dysfunction (ED) and atherogenesis can induce vascular cell apoptosis [10]. However, there is some *in vivo *evidence for a pro-atherogenic effect of apoptosis. A study in monkeys revealed that vascular ED was present without evidence of atherosclerosis, which may be due to endothelial apoptosis [11]. Apoptotic vascular cells are also found in hypercholesterolemic pigs and mice [12]. On the other hand, shear stress leads to physiologic low concentrations of nitric oxide (NO) within ECs [13]. The continuous generation of NO can prevent ECs apoptosis, thereby protecting the endothelial monolayer from injury [14]. Intervention with NO donor; L-arginine, has induced beneficial effects on atherosclerosis [15]. These findings strongly support the current clinical concept that ED precedes plaque formation and disease progression in patients [16].

The role of L-arginine and NO in apoptosis have been studied in different conditions (17–30). NO has also been demonstrated to be involved in the regulation of apoptosis, and recent evidence indicates that NO is a potent modulator of homeostasis operationally preventing or inducing apoptosis [31,32]. It is also reported that in some cell types, NO can promote apoptosis, whereas in others it inhibits apoptosis [33]. L-arginine as a NO donor is a potent substance to reverse ED [34-37]. Otsuji et al. studied the relationship between L-arginine and the progression of atherosclerosis. They found that exogenous L-arginine reverses acetylcholine-induced vasoconstriction in human coronary arteries in the early stages of atherosclerosis [38]. In hypercholesterolaemic rabbits treated with L-arginine, platelet aggregation, myointimal cell proliferation and vascular monocyte accumulation were attenuated while endothelium dependent vasoreactivity was improved [39]. Therefore, it is a hypothesized that L-arginine prevents ED through EC apoptosis inhibition in a model of hypercholesterolemia, and this hypothesis was tested in this study.

## Results

### Cholesterol and Nitrite Concentrations

The data for the cholesterol and nitrite concentrations are tabulated in table 1. The statistical analyses indicate that no significant difference was observed between the cholesterol levels of two groups, but the nitrite concentration in L-arginine group was significantly higher than control group (*p *< 0.05).

**Table 1:** The mean of serum cholesterol, LDL and nitrite levels in two groups of experiments.

group	cholesterol (mg/dl)		nitrite (μmol/l)	
	
	before	after	before	after
Control (n = 16)	111.7 ± 14.1	2129.1 ± 176.2	10 ± 0.7	11.8 ± 1
L-arginine (n = 14)	125.4 ± 14.5	2109.1 ± 166.9	11.6 ± 0.5	14.7 ± 0.5
p	>0.05	>0.05	>0.05	<0.05

### Fatty streak formation

The score of aorta fatty streak in first group was 0.875 ± 0.35, but no fatty streak lesion was detected in L-arginine group. The statistical analysis indicates that fatty streak formation is significantly lower in L-arginine group (*p *< 0.05).

### The number of intimal apoptotic cells

**Figure 1:**
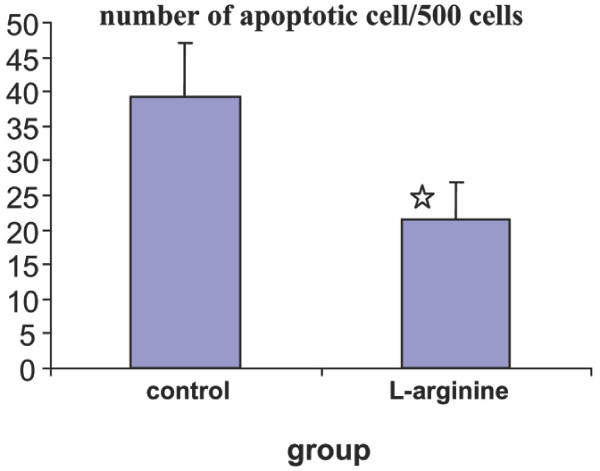
The intimal apoptotic cells in rabbit's aortas in two groups of animals. Figure shows that less apoptosis cells were observed in the aorta of L-arginine group. ☆ Indicate significant difference from control group (p < 0.05).

**Figure 2:**
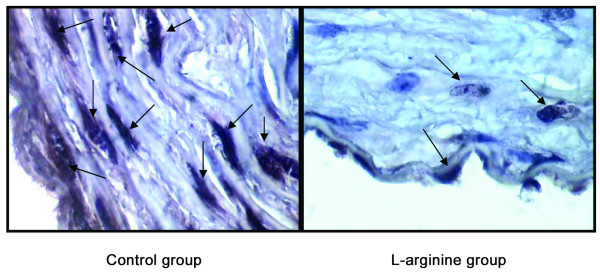
TUNEL performed in rabbit's aorta. Aorta section from rabbit that apoptosis of arterial cells is a prominent feature of atherosclerotic lesions (control group), but less apoptotic cells were observed in the aorta of L-arginine group.

The number of intimal apoptotic cells/500 cells in rabbit's aortas is demonstrated in figure 1 (control group: 39.3 ± 7.6, L-arginine group: 21.5 ± 5.3) (*p *< 0.05). In situ detection of apoptotic cells indicate that in aorta section from control group, the apoptosis of intimal cells is a prominent feature of atherosclerotic lesions, but less apoptosis cells were observed in L-arginine group (figure 2).

## Discussion

The role of L-arginine in ED prevention through EC apoptosis inhibition was the main objective of this study. There was no fatty streak in the aorta of L-arginine treated group while significant fatty streak lesions were found in control group. Subsequently, L-arginine supplementation successfully prevented atherosclerosis process, and this is in agreement with the results of several other studies in which L-arginine supplementation restores endothelial function [40-47].

Among the NO metabolites, nitrite is a major oxidative metabolite, which was implicated to be both an indicator for NO synthase (NOS) activity [48,49] and a circulating NO donor [50]. It has been shown that up to 70–90% of plasma nitrite derived from eNOS activity in fasted humans and other mammals [49,50].

The assumption of L-arginine efficacy theoretically has been based on eNOS activation and nitrite production enhancement. Our results were in line of this assumption, in which L-arginine supplementation led to significantly higher plasma nitrite concentration. Other studies have reported NO metabolites increasing in hypercholesterolemic animals [51-53]. Although decreased NO bioactivity (stems from flow mediated dilation studies) has been attributed to ED [54], increased plasma level of nitrite has been reported in hypercholesterolemic patients too [55]. It has been suggested that enhanced NO synthesis might be a defense mechanism to compensate continuous inactivation of NO and to protect from damaging factors such as hypercholesterolemia [56,57]. Another proposed mechanism for the elevation of nitrite may be NO production by other isoforms of NOS enzymes [57,58]. Although decreased activity of eNOS has been indicated in atherosclerosis but NO may be produced by iNOS in macrophages and other cell types in the atherosclerosis [57,58].

NO is also an essential signaling molecule for endothelial integrity and growth [59]. A moderate basal NO production can protect ECs from damaging effects of risk factor [49]. We hypothesized that if intrinsic protective mechanisms could be activated by moderate NO production in ECs, these cells could be better prepared to the ensuing risk factor (hypercholesterolemia) assault. Our results corroborate the protective role as the L-arginine group had significantly lower apoptotic cells in aorta intimal layer after 4 weeks of diet consumption. The precise mechanism responsible for inhibition of apoptosis by NO is not clear. Several possibilities exist that may explain the anti-apoptotic effects of NO. NO has been shown to increase Bcl-2, thioredoxin, and heat-shock protein-70 and -32 expression, and therefore it inhibits the release of mitochondrial cytochrome *c *and apoptosis inducing factors [33,60,61]. The activation of cGMP and cGMP-dependent protein kinase by NO increases a major intracellular anti-apoptotic protein, both directly and indirectly [62].

Also, it has been shown that NO inhibits the caspases-3 and 8 activations in L-arginine treated ECs and consequently inhibits apoptosis, which is consistent with our findings [63-65]. It should be further acknowledged, however, that the protective effect of L-arginine could also be mediated through non-eNOS-dependent pathways, since L-arginine has anti oxidant effects too [66,67]. Of course more studies are warranted in this field, and for future researches, using different doses of L-arginine and cholesterol diet in acute and chronic models of hypercholesterolemia are suggested.

## Conclusion

L-arginine attenuates the number of apoptosis cells in the aorta of a model of hypercholesterolemia. The inhibition of EC apoptosis may be the underlying mechanism of restore endothelial function by L-arginine.

## Methods

### Animals and Experimental design

This study was reviewed and approved by the Ethics Committee of Isfahan University of Medical Sciences. Thirty white male rabbits weighing 1.95 ± 0.25 kg were obtained from the Pasteur Institute of Iran. All animals were housed three per cage with free access to food and water. After 1-week acclimation period and an overnight fasting, blood samples were taken as pre-experimental sampling to obtain baseline data. Collected blood samples were centrifuged (10,000 _ *g*), and the resulting serum was stored at -70°C until measurements. The animals were then randomly assigned to 2 groups. The rabbits were fed rabbit chow supplemented with 1% cholesterol (hypercholesterolemic diet; control group, n = 16) or high-cholesterol diet with oral L-arginine (3% in drinking water) (L-arginine group, n = 14) for 4 weeks. Pure cholesterol and L-arginine were obtained from Scharlau Chemie (Barcelona, Spain) and Ajinomoto Co (Japan) respectively. At the end of experiment, fasting blood samplings were obtained, and half of the animals of each group randomly were selected and euthanized by an overdose of sodium pentobarbital (50 mg/kg) and ex-sanguinated. The animal's aortas were harvested for pathological investigation. The serum levels of cholesterol, and nitrite were measured. The fatty streak formation and the number of apoptotic cells also were determined as previously described [53,68,2,3].

### Serum cholesterol and nitrite measurements

Total cholesterol level was measured using standard enzymatic kit (Pars Azmoon Co, Iran). The serum level of nitrite (stable NO metabolite) was measured using a colorimetric assay kit (R&D Systems, Minneapolis, USA) that involves the Griess reaction. Briefly, serums were added into wells (96-well enzymatic assay plate). A sulphanilamide solution was added to all experimental samples, and after incubation, *N*-1-naphtylethylenediamine dihydrochloride solution was added. Then, absorbance was measured by a microreader in 540 nm wavelength. The samples nitrite concentration was determined by comparison to nitrite standard reference curve. The detection limit was 0.25 μM nitrite.

### Fatty streak determination

The abdominal aortas were subjected to pathological investigation to verify fatty dot or fatty streak lesions formation. The entire aorta, from the aortic arch to the external iliac arteries, was dissected out and cleaned of excess adventitial tissue. The aortas were fixed in buffered 10% formalin for 24 h, and then embedded in paraffin. The paraffin-embedded specimens were sectioned at 5 μm (20 sections in succession) and stained with haematoxylin and eosin, and examined by light microscopy to measure fatty streak by two pathologists in a double-blinded manner.

Fatty streak lesions were graded as zero for no fatty streak, 1 for existence of fatty streak in 1–4 sections, 2 for existence of fatty streak in 5–9 sections, 3 for existence of fatty streak in 10–14 sections and 4 for existence of fatty streak in 15 to all 20 sections of vessels.

### In situ detection of apoptotic cells by TUNEL method

The Terminal deoxynucleotidyl Transferase Biotin-dUTP Nick End Labeling (TUNEL) method was used for in situ detection of apoptotic cells by in situ cell death detection kit (Roche Applied Science, Indianopolis, IN, USA) as the manufacturer's instructions. Breifly, after dewaxation of formalin-fixed tissue sections; the slides were placed in a plastic jar containing 200 ml 0.1 M citrate buffer, pH 6.0, and were heated applying 350 W microwave irradiation for 5 min. After rinsing the slide with PBS (20°–25°C), they were immersed in a blocking solution containing 0.1 M Tris-HCl, 3% BSA, and 20% normal bovine serum, pH 7.5 for 30 min at room temperature, and washed again with PBS. Then TUNEL reaction mixture was added and incubated for 60 min at 37°C in a humidified chamber. The slides were washed, and anti fluorescein conjugated with alkaline phosphatase were added, and incubated again for 30 min. After rinsing in PBS, BCIP-NBT substrate solution was added and incubated for 15 min. The slides were subjected to wash extensively in tap water and were counterstained with hematoxylin. For apoptotic cells enumeration, at least 500 intimal cells were counted and the number of apoptotic cells was determined per 500 cells using light microscope.

### Statistical Analysis

The data are reported as the mean ± SEM. A statistical software package, SPSS (version 13), was used to perform statistical analysis. The data were tested for normality and homogeneity of variance. Otherwise, unpaired Student's *t*-test (equal or unequal variance assumed accordingly) was used to assess the significance of any change between groups. Statistical significance was accepted at *p *< 0.05.

## Abbreviations

EC: Endothelial Cell; ED: Endothelial Dysfunction; NO: Nitrix Oxide; NOS: Nitric Oxide Synthase; eNOS: Endothelial Nitric Oxide Synthase;  TUNEL: Terminal deoxynucleotidyl Transferase Biotin-dUTP Nick End Labeling.

## Competing interests

The authors declare that they have no competing interests.

## Authors' contributions

MN carried out the design and coordinated the study, participated in most of the experiments and prepared the manuscript. SH provide assistance in the design of the study, coordinated and carried out all the experiments and participated in manuscript preparation. FM and ALM provides assistance for all experiments. All authors have read and approved the content of the manuscript.
